# When a Pulmonary Nodule Mimics Malignancy: Primary Granular Cell Tumor of the Lung

**DOI:** 10.3390/diagnostics16101477

**Published:** 2026-05-13

**Authors:** Federica Pezzuto, Martina Maione, Chiara Giraudo, Marta Sbaraglia, Angelo Paolo Dei Tos, Fiorella Calabrese

**Affiliations:** 1 Department of Cardiac, Thoracic, Vascular Sciences, and Public Health, University of Padova, 35128 Padua, Italy; 2 University Hospital of Padova, 35128 Padua, Italy; 3 Department of Medicine, University of Padova, 35128 Padua, Italy

**Keywords:** granular cell tumor, lung, pulmonary nodule

## Abstract

Pulmonary nodules detected in patients with a history of malignancy are often clinically presumed to represent metastatic disease until proven otherwise. Granular cell tumor (GCT) is an uncommon, usually benign neoplasm of presumed Schwannian origin, which rarely occurs in the lung. Our aim is to emphasize the diagnostic challenges and the crucial role of histopathology in preventing overtreatment in oncology patients. Herein, we report the case of a 56-year-old woman with a previous history of papillary renal cell carcinoma diagnosed one year earlier, staged as pT1, WHO/ISUP grade 2, and treated with partial nephrectomy, with no evidence of residual disease or distant metastases at follow-up. During routine surveillance, she developed a solitary pulmonary nodule. Chest computed tomography (CT) showed a 12 mm solid nodule in the left upper lobe which was then further investigated with a positron emission tomography with 2-[^18^F] fluoro-2-deoxy-D-glucose [(^18^F)-FDG PET/CT, revealing a low glucidic uptake (SUVmax 4 and SUV mean 1.4). Endobronchial ultrasound-guided biopsy was non-diagnostic. Given the patient’s oncological history, the solid appearance on CT, and the mild FDG uptake, metastatic disease could not be excluded, and a parenchyma-sparing diagnostic wedge resection was performed. Histology showed a well-circumscribed proliferation of epithelioid cells with abundant granular eosinophilic cytoplasm and bland nuclei. Immunohistochemistry demonstrated diffuse S100 and CD68 positivity, supporting the diagnosis of primary pulmonary granular cell tumor. This case underscores the critical role of histopathological evaluation in the assessment of solitary pulmonary nodules, emphasizing that lesions identified during oncologic surveillance are not invariably indicative of malignancy.

**Figure 1:**
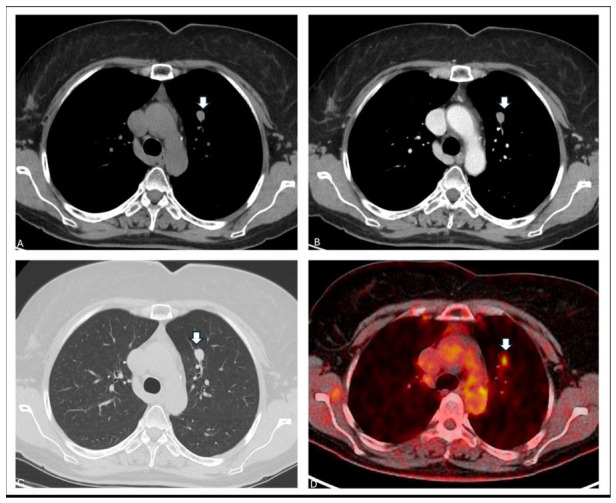
Pulmonary GCT is rare and has historically been estimated to account for approximately 6–10% of all GCTs. Within the respiratory tract, it most frequently arises in the tracheobronchial tree rather than in the peripheral lung parenchyma [1,2]. Endobronchial lesions typically present with symptoms related to airway obstruction, including recurrent pneumonia, hemoptysis, or persistent atelectasis [3]. Large clinical series have shown that a substantial proportion of tracheobronchial GCTs are discovered incidentally and that coexistence with other malignancies is not uncommon [4,5]. Radiologically, pulmonary GCT lacks specific features and may present as a well-defined nodule or endobronchial mass; in oncological patients, as in our case (Figure 1), this appearance may raise concern for metastases. In particular, metastases from papillary renal cell carcinoma usually appear as round lesions with smooth margins and their FDG uptake can be variable [6]. Similarly, FDG uptake in granular cell tumor is variable and may overlap with both benign and malignant lesions [7]. Advanced imaging techniques like radiomics, favoring the distinction between benign and malignant nodules—including metastases—might be applied in the future in similar circumstances providing additional information [8,9]. In the present case, a contrast-enhanced chest computed tomography (CT) was performed during routine oncologic follow-up for papillary renal cell carcinoma in a 56-year-old woman, which revealed a solid peribronchovascular pulmonary nodule in the anterior segment of the left upper lobe. The chest CT nicely showed the 12 mm nodule with smooth margins and homogeneous contrast enhancement in the left upper lobe (white arrows) ((**A**): axial mediastinal window with contrast; (**B**): axial mediastinal window without contrast; (**C**): lung window). The nodule did not show any contact with the pleura, cavitation or calcification. The axial fused [^18^F] FDG/PETCT (Siemens Biograph mCT 20 Excel; no further technical information was available due to the retrospective study design) showed mild tracer uptake (SUVmax 4 and SUVmean 1.4) of the nodule (white arrow) (**D**).

**Figure 2:**
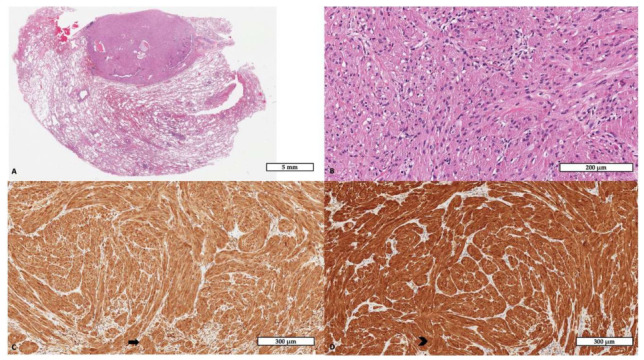
Histological examination of the wedge resection specimen revealed a well-circumscribed proliferation within the lung parenchyma ((**A**), hematoxylin and eosin, scale bar: 500 µm). At higher magnification, the tumor was composed of polygonal epithelioid cells arranged in nests and cords, characterized by abundant granular eosinophilic cytoplasm and round nuclei without significant atypia or mitotic activity ((**B**), hematoxylin and eosin, scale bar: 200 µm). Immunohistochemistry demonstrated strong and diffuse cytoplasmic and nuclear positivity for S100 protein ((**C**), scale bar: 300 µm; the black arrow indicates a representative area of positive staining). CD68 showed granular cytoplasmic staining, highlighting the lysosome-rich nature of the tumor cells ((**D**), scale bar: 300 µm; the black arrowhead indicates a representative area of positive staining). An extended immunohistochemical panel was performed for differential diagnostic purposes. Pancytokeratin, TTF1, STAT6, HMB45, MelanA, SMA, and desmin were negative, excluding epithelial, solitary fibrous tumor, melanocytic/PEComa-like, and smooth muscle differentiation. Pulmonary GCT is a neoplasm showing Schwannian differentiation, characterized by sheets of polygonal cells with lysosome-rich cytoplasm and diffuse S100 expression [10]. CD68 positivity reflects intracytoplasmic lysosomal accumulation and should not be misinterpreted as histiocytic differentiation. The absence of pancytokeratin helps exclude metastatic carcinoma, while the lack of STAT6 expression rules out solitary fibrous tumor [11]. Among other uncommon pulmonary lesions entering the differential diagnosis, inflammatory myofibroblastic tumor may also present as a solitary mass-like lesion and mimic malignancy, but it usually shows a spindle-cell myofibroblastic proliferation with a prominent inflammatory background and, in a subset of cases, ALK expression or rearrangement [12]. The diagnostic work-up of uncommon pulmonary tumors is often complex. This difficulty is further increased in small biopsy specimens, where limited sampling may obscure the defining morphological features. Therefore, accurate diagnosis requires careful correlation between histology, immunohistochemistry, and the clinical–radiological context [13]. Although the vast majority of pulmonary GCTs are benign, rare malignant cases have been described [14]. Histologic criteria for malignancy include necrosis, increased mitotic activity, spindling, pleomorphism, prominent nucleoli, and high nuclear-to-cytoplasmic ratio [15]. None of these adverse features were present in the current case. Molecular data specific to pulmonary GCT remain limited [2,16,17]. Genomic studies of GCTs across different anatomical sites have identified recurrent alterations in genes encoding components or regulators of the V-ATPase pathway, particularly ATP6AP1 and ATP6AP2, supporting V-ATPase dysfunction as a relevant mechanism in GCT tumorigenesis [18,19]. However, no consistent site-specific molecular profile has yet been defined for pulmonary GCTs. Management depends on tumor location and clinical suspicion. Centrally located lesions may be treated endoscopically or surgically depending on size and depth of invasion [5,20]. In contrast, peripheral nodules in oncologic patients are frequently resected because of preoperative concern for metastasis [21]. Other rare pulmonary mesenchymal tumors, such as solitary fibrous tumor, may also present as solitary lesions; in these cases, complete surgical excision with negative margins remains the treatment of choice, followed by long-term surveillance because of the potential risk of recurrence [22]. More broadly, when a pulmonary lesion is suspected to be malignant, the surgical approach should be tailored to tumor extent and anatomical involvement [23]. Although rare, primary pulmonary granular cell tumor should be considered in the differential diagnosis of solitary pulmonary nodules detected during oncologic surveillance. Despite radiological features that may mimic metastases, definitive histopathological evaluation is crucial to avoid misclassification and overtreatment.

## Data Availability

The original contributions presented in this study are included in the article. Further inquiries can be directed to the corresponding author.

## Abbreviations

The following abbreviations are used in this manuscript:

GCT	Granular Cell Tumor
CT	Computed Tomography
PET	Positron Emission Tomography
^18^F-FDG	^18^F-Fluorodeoxyglucose
SUVmax	Maximum Standardized Uptake Value
H&E	Hematoxylin and Eosin

## References

[1] Deavers M., Guinee D., Koss M.N., Travis W.D. (1995). Clinicopathologic Study of 20 Cases. Am. J. Surg. Pathol..

[2] Houcine Y., Mlika M., Moussa C., Rouis H., Brahem E., Ismail O., Maȃlej S., El Mezni F. (2023). Granular Cell Tumor of the Lung and Tracheobronchial Tree: Two Case-Presentation with a Review of the Literature. Rare Tumors.

[3] Hernandez O.G., Haponik E.F., Summer W.R. (1986). Granular Cell Tumour of the Bronchus: Bronchoscopic and Clinical Features. Thorax.

[4] Van der Maten J., Blaauwgeers J.L.G., Sutedja T.G., Kwa H.B., Postmus P.E., Wagenaar S.S. (2003). Granular Cell Tumors of the Tracheobronchial Tree. J. Thorac. Cardiovasc. Surg..

[5] Lachkar S., Roger M., Vergnon J.M., Crutu A., Febvre M., Mehdaoui A., Gervais R., Geriniere L., Arbib F., Dayen C. (2023). Endoscopic Resection of Tracheal and Bronchial Granular Cell Tumours: A National Multicentre Retrospective Study. Respirology.

[6] Griffin N., Gore M.E., Sohaib S.A. (2007). Imaging in Metastatic Renal Cell Carcinoma. Am. J. Roentgenol..

[7] Cheng A.-P., Dong M.-J., Fu L.-P., Zhao L.-J., Wang X.-G., Sun W.-Y. (2014). A Rare Case of Pulmonary Malignant Granular Cell Tumor Detected with 18 F-FDG PET/CT Imaging. Clin. Nucl. Med..

[8] Warkentin M.T., Al-Sawaihey H., Lam S., Liu G., Diergaarde B., Yuan J.M., Wilson D.O., Atkar-Khattra S., Grant B., Brhane Y. (2024). Radiomics Analysis to Predict Pulmonary Nodule Malignancy Using Machine Learning Approaches. Thorax.

[9] Cicchetti G., Marano R., Strappa C., Amodeo S., Grimaldi A., Iaccarino L., Scrocca F., Nardini L., Ceccherini A., Del Ciello A. (2025). New Insights into Imaging of Pulmonary Metastases from Extra-Thoracic Neoplasms. Radiol. Medica.

[10] Ordóñez N.G. (1999). Granular Cell Tumor: A Review and Update. Adv. Anat. Pathol..

[11] International Agency for Research on Cancer (IARC) (2021). WHO Classification of Tumours of the Lung, Pleura, Thymus and Heart.

[12] Vounckx M., Jansen Y.J.L., Fadaei S., Geers C., De Pauw V., Smets D. (2024). Unraveling the Spectrum of Inflammatory Myofibroblastic Tumors in the Lung: A Comprehensive Case Series Highlighting Endobronchial, Pleural, and Lung Parenchymal Tumors. JTCVS Open.

[13] Moran C.A. (2025). Uncommon Tumors of the Lung: Recently Described and Rediscovered Tumors. Arch. Pathol. Lab. Med..

[14] Jiang M., Anderson T., Nwogu C., Tan D. (2003). Pulmonary Malignant Granular Cell Tumor. World J. Surg. Oncol..

[15] Fanburg-Smith J.C., Meis-Kindblom J.M., Fante R., Kindblom L.-G. (1998). Malignant Granular Cell Tumor of Soft Tissue. Am. J. Surg. Pathol..

[16] Zhang H., Hu F., Zhang X., Zhang Y., Li C., Chen Y., Li F. (2020). Clinicopathological Characteristics and Gene Analysis of Pulmonary Granular Cell Tumor in Three Cases and a Systematic Review. Transl. Cancer Res..

[17] Torrado C., Camaño M., Hindi N., Ortega J., Sevillano A.R., Civantos G., Moura D.S., Dimino A., Martín-Broto J. (2023). Antiangiogenics in Malignant Granular Cell Tumors: Review of the Literature. Cancers.

[18] Sekimizu M., Yoshida A., Mitani S., Asano N., Hirata M., Kubo T., Yamazaki F., Sakamoto H., Kato M., Makise N. (2019). Frequent Mutations of Genes Encoding Vacuolar H^+^-ATPase Components in Granular Cell Tumors. Genes Chromosom. Cancer.

[19] Pareja F., Brandes A.H., Basili T., Selenica P., Geyer F.C., Fan D., Da Cruz Paula A., Kumar R., Brown D.N., Gularte-Mérida R. (2018). Loss-of-Function Mutations in ATP6AP1 and ATP6AP2 in Granular Cell Tumors. Nat. Commun..

[20] Ishibashi H., Baba S., Nakashima Y., Takasaki C., Kobayashi M., Okubo K. (2018). Endobronchial Granular Tumor Excision with Bronchial Resection Inclusive of Second Carinoplasty. Ann. Thorac. Surg..

[21] Al-Ghamdi A.M., Flint J.D.A., Muller N.L., Stewart K.C. (2000). Hilar Pulmonary Granular Cell Tumor: A Case Report and Review of the Literature. Ann. Diagn. Pathol..

[22] Ajouz H., Sohail A.H., Hashmi H., Martinez Aguilar M., Daoui S., Tembelis M., Aziz M., Zohourian T., Brathwaite C.E.M., Cerfolio R.J. (2023). Surgical Considerations in the Resection of Solitary Fibrous Tumors of the Pleura. J. Cardiothorac. Surg..

[23] Dell’Amore A., Campisi A., Bertolaccini L., Chen C., Gabryel P., Ji C., Piwkowski C., Spaggiari L., Fang W., Rea F. (2022). A Multicenter Retrospective Cohort Study on Superior Vena Cava Resection in Non-Small-Cell Lung Cancer Surgery. Cancers.

